# Comparative Immunohistochemical Analysis of Ochratoxin A Tumourigenesis in Rats and Urinary Tract Carcinoma in Humans; Mechanistic Significance of p-S6 Ribosomal Protein Expression

**DOI:** 10.3390/toxins4090643

**Published:** 2012-09-11

**Authors:** Patrycja Gazinska, Diana Herman, Cheryl Gillett, Sarah Pinder, Peter Mantle

**Affiliations:** 1 Breakthrough Breast Cancer Research Unit, Guy’s Hospital, King’s Health Partners AHSC, King’s College London School of Medicine, London SE1 9RT, UK; Email: patrycja.gazinska@kcl.ac.uk; 2 Breast Tissue and Databank, Guy’s Hospital, King’s Health Partners AHSC, King’s College London School of Medicine, London SE1 9RT, UK; Email: cheryl.gillett@kcl.ac.uk; 3 Pathology Department, County Hospital Timisoara, Timisoara 300736, Romania; Email: diaherman@yahoo.com; 4 Department of Research Oncology, Guy’s Hospital, King’s Health Partners AHSC, King’s College London School of Medicine, London SE1 9RT, UK; Email: sarah.pinder@kcl.ac.uk; 5 Centre for Environmental Policy, Imperial College London, London SW72AZ, UK

**Keywords:** ochratoxin A, phospho-S6 ribosomal protein, mycotoxin, renal cell carcinoma, transitional cell carcinoma, testicular cancer, angiosarcoma, Balkan endemic nephropathy, food safety, DNA adducts

## Abstract

Ochratoxin A (OTA) is considered to be a possible human urinary tract carcinogen, based largely on a rat model, but no molecular genetic changes in the rat carcinomas have yet been defined. The phosphorylated-S6 ribosomal protein is a marker indicating activity of the mammalian target of rapamycin, which is a serine/threonine kinase with a key role in protein biosynthesis, cell proliferation, transcription, cellular metabolism and apoptosis, while being functionally deregulated in cancer. To assess p-S6 expression we performed immunohistochemistry on formalin-fixed and paraffin-embedded tumours and normal tissues. Marked intensity of p-S6 expression was observed in highly proliferative regions of rat renal carcinomas and a rare angiosarcoma, all of which were attributed to prolonged exposure to dietary OTA. Only very small OTA-generated renal adenomas were negative for p-S6. Examples of rat subcutaneous fibrosarcoma and testicular seminoma, as well as of normal renal tissue, showed no or very weak positive staining. In contrast to the animal model, human renal cell carcinoma, upper urinary tract transitional cell carcinoma from cases of Balkan endemic nephropathy, and a human angiosarcoma were negative for p-S6. The combined findings are reminiscent of constitutive changes in the rat tuberous sclerosis gene complex in the Eker strain correlated with renal neoplasms, Therefore rat renal carcinogenesis caused by OTA does not obviously mimic human urinary tract tumourigenesis.

## 1. Introduction

Ochratoxin A is well known as one of the first mycotoxins to be discovered in the 1960s, later shown to be responsible for chronic nephropathy in commercial pig production [1], and as generally toxic in experimental animals [2]. Its specific significance arose when it was shown to be a potent renal carcinogen in male rats after protracted exposure via oral gavage, which was generally well tolerated [3,4]. Feeding studies with male mice extended the range of OTA carcinogenicity [5] and in subsequent rat carcinogenesis studies the toxin was also homogenised into diet [6]. 

Suspected relevance for humans still relates to natural dietary contamination, although very small amounts of toxin occur occasionally in some agricultural commodities. Historically, some pig products were a potential source until the risk was recognised. It is also possible that the wide sensitivity of animals to the toxin shown in experimental toxicology can be extended to man, but this remains unclear [2]. Nevertheless, particular focus on ochratoxin A as a renal carcinogen remains. The fact that most human renal carcinoma remains idiopathic [7] leaves ample room for hypothetical aetiologies. Potential adverse impact on food industries, particularly those using cereals (generically) and coffee or cocoa (specifically), mainly concerned marketing and product and brand image. However, some relief was found in the statutory differentiation amongst chemical carcinogens between those that are demonstrably genotoxic by binding to DNA and those that only act via indirect mechanisms. Currently there is no agreed mechanism of OTA carcinogenesis in the rat and it is even unclear whether a rat model is relevant for human upper urinary tract carcinoma. Humans would have to be particularly sensitive to OTA for the common European average dietary intake of the order of 2–3 ng OTA/kg b.w. [2] to ever exceptionally and consistently match the 15 µg/kg b.w. tumourigenic threshold demonstrated for rats during continuous lifetime gavage exposure in the NTP study [3], or even to the 20–30 µg/kg body weight value for dietary exposure [8]. 

Meanwhile, to assess validity of a rat model for human risk assessment, the opportunity of having archived tissues from rat lifetime carcinogenicity studies [6,9,10,11,12] has been taken to study genetic change in OTA-generated rat tumours. To date no mutations in genes associated with human familial renal cell carcinoma have been detected [13]. However, since inactivation of the *Tsc2* and folliculin genes in mice have been associated with the development of renal tumours and mammalian target of rapamycin (mTOR) disregulation [14], we investigated the OTA-associated tumours for evidence of mTOR activation. A few rare human cases of familial renal cell carcinoma are also attributable to disruption of the *TSC2* gene [15]. 

mTOR is involved in the regulation of S6 kinase activity and subsequent phosphorylation of the ribosomal protein S6. Deregulation of S6 phosphorylation could contribute to tumorigenesis by activation of the PI3K signal-transduction pathway in cancer. The tumour suppressor tuberous sclerosis complex-2 (*TSC2*) and *TSC1*, acts as an antagonist of S6 kinase activation [16]. The S6 phospho-specific antibody was used in order to assess the activity of mTOR/TSC upon mycotoxin-induced tumours in rats and in some human urinary tract carcinoma. It is also an opportunity to validate an experimental rat model in predicting aetiology of some idiopathic human carcinogenesis.

## 2. Results

**Figure 1:**
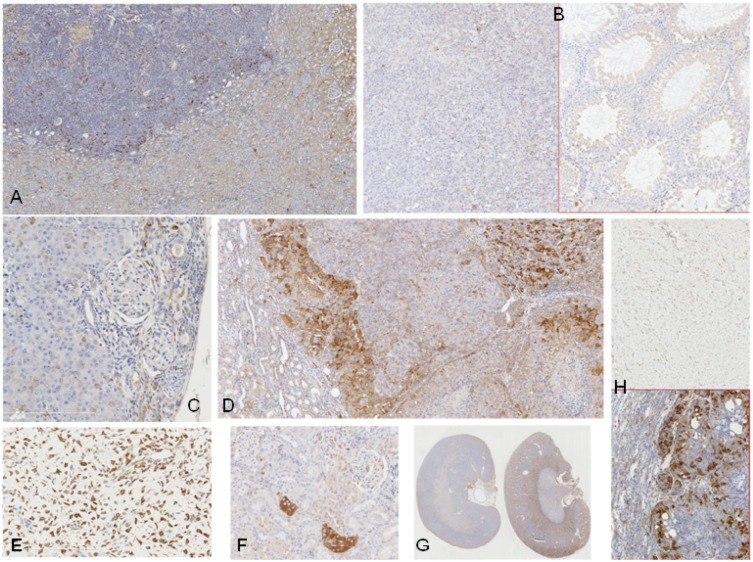
Contrasting findings on expression of p-S6 protein in renal, testicular and mammary tumours, and renal tissues, of rats treated with OTA. (**A**) (case 1) part of a small *in situ* renal tumour with engulfment of adjacent glomeruli and scattered foci staining intensely for p-S6 protein. (**B**) (case 5), consistent lack of p-S6 protein in both normal testicular seminiferous tubules (right) and in adjacent seminoma (left); (**C**) (case 3), similar absence of p-S6 protein in kidney cortex (right) and adenoma (left); (**D**) (case 7) tumour (right), partly adenoma (centre) partly surrounded by infiltrating carcinoma with p-S6 expression; contrast with normal kidney (left); (**E**) (case 4), mammary angiosarcoma with extensive evenly-distributed p-S6 positive elements; (**F**) (case 2), isolated intensely-stained elements in the non-tumour kidney (contralateral kidney had a tumour); (**G**) (cases 18 and 19), kidney after 6 months (left) and 16 months (right), showing changes in regional pattern revealed by p-S6 antibody; (**H**) (case 1), subcutaneous tumour (normal ageing pathology in occasional rats), above, unstained for p-S6 protein contrasting with the expression in adenoma-like regions of kidney tumour, below, but not in adjacent kidney.

**Figure 2:**
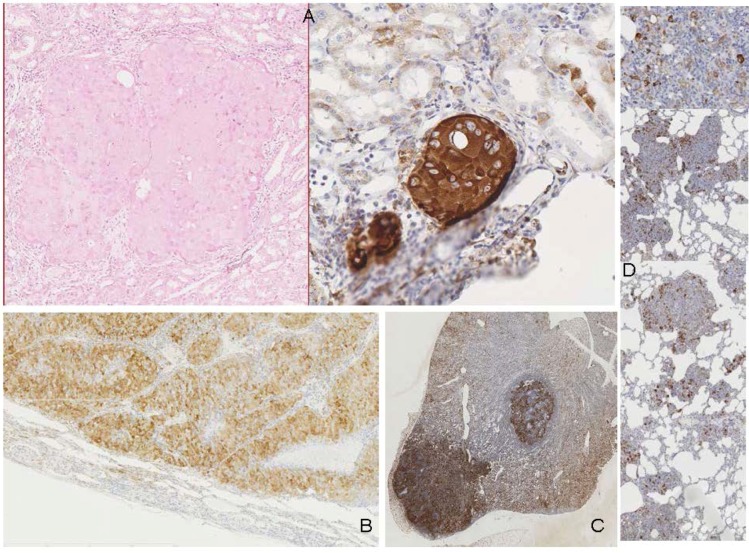
Recognition of OTA carcinogenicity in rats. (**A**) (case 17), H & E stained section of small *in situ* renal carcinoma (left), matched with position (magnified) of tumour edge elements stained intensely for p-S6 protein in another section from the same block; (**B**) (case 12, also Figure 4), renal tumour edge (above), stained for p-S6, contrasting with unstained stretched kidney cortex with distorted glomerulus (below); (**C**) (case 3), carcinoma, intensely stained for p-S6 into regions of kidney sectioned through the papilla (top right); (**D**) (case 1), lung tissue, from rat with renal carcinoma, showing scattered intensely-stained elements, more clearly seen in magnified region and attributed to metastatic fragments from primary renal tumour.

**Figure 3:**
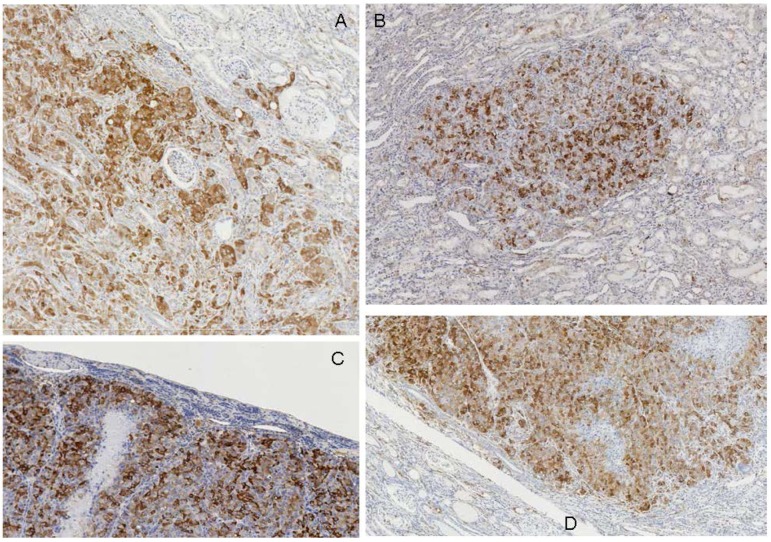
Immunohistochemical demonstration of marked expression of p-S6 protein in renal tumours of rats treated with OTA. (**A**) (case 5), contrasting junction between tumour periphery, including cortical glomeruli engulfed by invasive carcinoma, and unstained adjacent renal cortex; (**B**) (case 10), carcinoma within kidney; (**C**) (case 12), periphery of large compression tumour stretching encompassing cortex; (**D**) (case 11), carcinoma contrasting with surrounding renal tissue.

**Figure 4:**
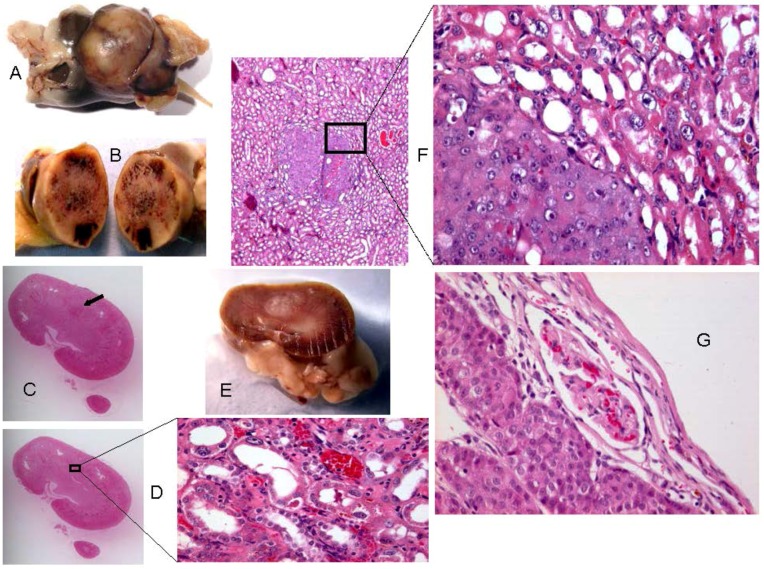
Renal tumour pathology (case 12, Supplementary Table S1) of the largest compression tumour found in all ochratoxin A (OTA) studies at Imperial College [6,8,10,11,12] illustrating adenoma histology. (**A**) formalin-fixed tumour, splitting functional (right) and necrotic (left) kidney tissue to opposite poles, with associated peri-renal fat. (**B**) divided spherical adenoma showing areas of haemorrhage. (**C**) section (H & E stained) from functional kidney showing small tangential tumourfragment (arrow). (**D**) section just beyond tumour edge, with expanded detail showing karyomegaly diagnosing outer medulla. (**E**) central tumour in formalin-fixed kidney, (**F**) separate micro-tumour with expanded detail of tumour-kidney junction; carcinoma bottom left, medulla with karyomegaly top right. (**G**) main tumour edge showing adenoma (left) bounded by stretched kidney cortex with distorted glomerulus (see immunohistochemical staining in Figure 2B).

The following descriptions of immunohistochemical findings in tissues are best read in conjunction with rat case context detail and literature cross-referencing tabulated in Supplementary Data. Findings are also summarised in Table 1 and illustrated in Figure 1, Figure 2, Figure 3, Figure 4.

### 2.1. Rat Controls

Kidney sections of neonatal and young adult male rats (Supplementary Table S1, cases 20 and 21), not intentionally exposed to OTA, generally showed no staining via the p-S6 antibody. Occasional scattered diffuse light-brown staining of some parenchymal elements occurred which may either indicate expression of p-S6 synthesis or just be a technical artefact. Some non-tumourous cortical regions of tumourous kidneys of rats given chronic exposure to dietary OTA showed similar diffuse features apparently indicative of S6 expression, but medullary tissues were consistently negative.

### 2.2. Rats Exposed Experimentally to OTA

Case 1. This old animal, having lost condition, was found to have a large (25 g) renal tumour, haemorrhaged to fill the abdomen with ascites. Extensive metastatic nodules were on serosal surfaces within the abdomen, but not within the thorax. The contralateral kidney appeared normal, but histology revealed a small carcinoma *in situ*. IHC showed small foci of p-S6 in the tumour (Figure 1A). Similar foci were distributed within tissue of lung (Figure 2D), implying that they were small metastatic fragments in an organ which seemed at necropsy, to have escaped cancer from the 6 months exposure to OTA.

Case 2. By comparison with case 1 above, renal neoplasms in response to a further three months of OTA exposure can be recognised by overexpression of p-S6. IHC highlighted a small adeno-carcinoma distinct from the surrounding tissue of one kidney, though not strongly. However, a small tubule element in the other kidney was intensely stained (Figure 1F). Notably, the rat was the oldest studied and necropsy was performed 83 weeks after OTA exposure ceased.

Case 3. Intense staining within most of the proliferated tubules of a renal tumour in two regions (Figure 2C), contrasted with two examples of homogeneous areas within a large subcutaneous sarcoma (one example, Figure 1H), implying a specific aetiological connection only between OTA and the renal tumour.

Case 4. This case differed from case 3 mainly by gender (female), but also, after OTA exposure, by the rat receiving sodium barbitate in drinking water for life at a dose known to be a promoter of tumours initiated by nickel [17]. Scattered stained elements were evident rather specifically in cortex, seen in both kidneys either in perpendicular or transverse renal sections. There had neither been histopathological evidence of renal tumour, nor did IHC detect any particular neoplastic foci. However, the animal had developed a large mammary tumour within which were regions recognised as angiosarcoma; the sarcomatoid elements were intensely stained throughout this region (Figure 1E).

Case 5. Intense staining occurred across regions of the renal tumour surrounding a necrotic centre, contrasting with absence of staining in the adjacent normal kidney (Figure 3A). Carcinoma arising during lifetime exposure to OTA had infiltrated into cortex, enclosing unstained glomeruli. A testis tumour in this rat was histologically typical of age-related seminoma and was homogeneously negative with IHC as were adjacent seminiferous tubules (Figure 1B).

Case 6. The infiltrating carcinoma was stained for p-S6 in contrast to the remaining kidney tissue.

Case 7. General intense staining of disorganised carcinoma across a small tumour *in situ*, but enclosing an area with adenoma histopathology in which the lack of staining was similar to that in the kidney (Figure 1D). Notably, typical rat seminomas in testis of the same animal were negative as in case 5; therefore they can not be attributed to the OTA exposure.

Case 8. This case was from a rat with bilateral renal tumours, the other being very large and metastatic with dissemination of nodules across abdominal serous surfaces. IHC of the smaller kidney clearly differentiated tumour, but staining was poor in the part most distal from the blood supply. 

Case 9. After a lifetime on the highest OTA intake, this rat’s large tumour arising from the cranial aspect of the right kidney contrasted with the perfect condition of the left kidney showing the highly focal occurrence of ochratoxin’s renal cancer.

Case 10. The tumour surrounded a large cyst and was composed of adenoma and carcinoma regions, parts of which (surrounding the cyst and elsewhere) contained elements that were intensely stained for p-S6. Distant from the tumours, two portions of a swollen tubule were also intensely stained (Figure 3B).

Case 11. Intense staining for p-S6 in large areas of infiltrating renal carcinoma contrasts with adjacent kidney parenchyma (Figure 3D).

Case 12. A large compression tumour with consistent adenoma histopathology throughout showed positive staining around the periphery within expanded tubule conformations, contrasting with the stretched cortex layer enclosing the whole tumour (Figure 2B, Figure 4).

Cases 13–16. These cases are the group that arose in a study on effects of chronic OTA exposure only in the second half of life [11], and in which small renal neoplasms were recognised in H & E stained sections. IHC showed that the first, a compression tumour centred apparently within the renal medulla, was virtually free of any elements stained for p-S6 and had a homogeneous adenoma histopathology (Figure 1C). The second (Figure 3C) and third similarly had a negative response in the micro-adenomas. The fourth concerned a larger tumour with general adenoma structure; stretched kidney cortex surrounding the tumour did not show evidence of p-S6, but peripheral parts of the tumour had scattered foci stained to imply presence of p-S6. This situation may indicate early progression towards adenocarcinoma in younger peripheral proliferations in this larger tumour, but it is reminiscent of the situation in the similarly-sized tumour of case 12 above.

Case 17. In this case, in which no overt renal tumour had been evident, a micro-carcinoma was recognised, located close to the innermost glomeruli at the cortico-medullary junction. Revisiting the original wax block for IHC revealed a tumour remnant with similar swollen tubule elements and stained intensely for p-S6 at the corresponding spacial coordinates (Figure 2A), thereby recognising profound genetic change implied for this tumour, arising from a lifetime’s low OTA dose regimen [12]. 

Cases 18 and 19. Arising from an experiment on a threshold, non-tumourigenic, exposure to OTA, IHC of kidney at the 30-week stage showed faint differentiation of cortical and medullary regions, but no specific evidence of p-S6. At the 75-week stage the regional differentiations seen at the 30-week stage were accentuated (Figure 1G). There were patches of diffuse staining in cortex, but virtually none in the outer medulla. Diffuse, non-specific staining was in the inner medulla, contrasting with very few isolated patches in the papillae.

### 2.3. Human Urinary Tract Tumours

Sections from wax blocks of four Romanian transitional cell carcinomas from the upper urinary tract of cases of Balkan endemic nephropathy, previously used for DNA ploidy distribution measurement (one shown to be diploid and the others aneuploid) [9,18], were completely negative for p-S6 protein. A section of a Danish renal cell carcinoma (diploid) [9] also gave a negative response, as did a Romanian human angiosarcoma. Reciprocal analysis of the angiosarcoma of rat case 4 in Romania showed that the tissue did not express the human proteins CD31, CD34 or D2-40. The consistent negative staining findings are not illustrated because they are the same as for the tumourous testis in Figure 1B. 

In summary (Table 1), overexpression of p-S6 protein synthesis was evident in all 11 rat renal carcinomas, diffusely or focally within each tumour, in contrast to its absence in three small renal adenomas. Evidence of the protein peripherally in two larger renal tumours may indicate that this tumour tissue close to kidney vasculature was already adenoma in transition towards carcinoma, even if not yet phenotypic of invasive carcinoma and therefore not previously recognised by conventional H & E staining. P-S6 was also absent in two testes with multiple seminomas and in a large subcutaneous tumour, natural tumours in ageing rats even those given OTA. However, the striking staining in the mammary angiosarcoma of rat case 4 suggested that OTA was involved in its etiology, even if in experimental association with sodium barbitate. Nevertheless, the striking occurrence of widespread, small stained foci, interpreted as metastatic from kidney primary carcinoma, in lung is a further indicator of the specificity of p-S6 in tissues with OTA-generated genetic change. Therefore the contrast with the consistent absence of p-S6 in all the human urinary tract carcinomas and an angiosarcoma is striking.

**Table 1:** Summary of comparative immunohistochemical findings for p-S6 in rat and human tumours.

	Pathological finding	Total cases	Positive	Negative
Rat	Kidney, carcinoma or adenocarcinoma	13	13	0
Rat	Lung, carcinomas (metastatic from kidney)	1	1	0
Rat	Kidney, small adenoma (*in situ*)	3	0	3
Rat	Mammary, angiosarcoma	1	1	0
Rat	Subcutaneous fibrosarcoma	1	0	1
Rat	Testis, seminomas	2	0	2
Human	Upper urinary tract, transitional cell carcinoma (Balkan endemic nephropathy)	4	0	4
Human	Kidney, renal cell carcinoma	1	0	1
Human	Angiosarcoma	1	0	1

## 3. Discussion

The rat tumour tissues were available from experiments on tumourigenic response to dietary OTA dose regimens, duration of exposure, latency, and both threshold and tolerable dosages. Tissues became available from old or ageing animals when euthanized due to clinical morbidity. No tissues were from decedents. Thus, tumours ranged from large aggressive metastatic carcinoma to cryptic micro-adenoma. It is assumed that in many cases the common developmental sequence from adenoma to carcinoma occurred, and that this may have involved additional genetic change, whether through direct genotoxic insult or through epigenetic influences. This is implied from the DNA ploidy distribution studies on these and other tissues [18] showing diploid adenoma and aneuploid carcinoma. Nothing is known about the kinetics of rat renal tumour growth in response to OTA, bearing in mind that at least six months of continuous exposure to contaminated feed seems necessary in the first year of life at a daily intake of ~0.3 mg/kg to be sure of causing renal cancer in some individuals. Further, tumours have rarely been discovered in animals less than 18 months old, there can be a year’s latency between ceasing OTA exposure and discovering a renal tumour, and no time-course experiments have been performed. The latter would be a major undertaking, comparable in magnitude to the NTP study [3], but modern knowledge could optimise yield of tumours in a hybrid rat [10]. In our experience, protracted OTA exposure that is well tolerated is likely only to cause unilateral renal tumourigenesis, probably from a single focus or at most very few foci. In larger renal carcinomas the precise point of origin has been obscured, but the smaller neoplasms tend to centre in the outer medulla close to innermost cortical glomeruli. Thus the present tumour material is unique, and necessarily heterogeneous both in OTA exposure and histology.

The human tumours for the present study were chosen because their histopathology and DNA ploidy distribution had all been studied previously [9] and ranged from diploid to marked aneuploidy lesions. 

The clinical context for interpreting the present findings from immunohistochemical examination of rat and human tumours is that phosphorylated S6 ribosomal protein is a marker indicating activity of the mammalian target of the anti-tumour drug rapamycin (mTOR) in cell proliferation. Expression of p-S6 might therefore be expected in some malignant tissues and, indeed, marked expression has been illustrated [19] in tumour cells lining some renal cysts in mice with mutation in the gene *Tsc1* that forms part of the tuberous sclerosis complex. Illustrations differentiated between staining for p-S6 protein in cells (~100 m^2^) lining one third of small (100–200 µm diameter) renal cysts. Staining was absent in others. However, intense staining occurred in >90% of renal cell carcinomas and cystadenomas, the cells of which were abnormally large (~180 µm^2^). 

Tuberous sclerosis complex is an autosomal dominant human syndrome with benign and occasionally malignant tumours in CNS, skin and kidney. Two human genes, *TSC1* and *TSC2*, are involved. The Eker rat, heterozygous for a dominantly inherited germline mutation in the *Tsc2* tumour suppressor gene, is recognised as a valid model for human tuberous sclerosis complex [20]. Intense illustrated immunohistochemical staining of renal tumour, contrasting with associated renal tissue, extends also in [19] to recognising a small cluster of cells in a renal tubule as a potential tumourous neoplasm. Notably, the latter situation seems to be matched in the present findings. Mutations in either the *Tsc1* or the *Tsc2* gene can cause the pathology of the tuberous sclerosis complex [19]. Wilson *et al* [18] generated *Tsc1*^+/−^ mice with predisposition to develop cysts and then to progress to cystadenoma and renal cell carcinoma. They then identified somatic *Tsc1* mutation in ~80% of these tumours but only in one third of cysts. A role for haploinsufficiency in *Tsc1* in cyst formation was proposed. Tumours showed much stronger staining for p-S6 protein than did the cysts. We therefore conclude that consistent overexpression of p-S6 protein in rat renal carcinomas caused by OTA in the present study implies modulation concerning mTOR signalling.

It is particularly notable where, within animals, p-S6 differentiated between “normal” rat tumours (testis seminoma or subcutaneous fibrosarcoma that were not stained) and ochratoxin-generated renal carcinomas that were stained. The complete absence of p-S6 staining in the three small renal adenomas found in rats over two years old (OTA given only in the second year) may be demonstrating that genetic or epigenetic events associated with mTOR pathway dysregulation is a later step associated with progression to carcinoma. However, the precise status of these small neoplasms as benign or having proliferating potential remains unclear.

Strong diffuse staining for p-S6 protein has also been shown in some human soft tissue sarcomas [21]. The present finding of consistent overexpression of p-S6 protein in a rare rat mammary angiosarcoma associated with OTA exposure, suggests that genetic changes targeted by OTA also caused this tumour, potentially via change in the *Tsc1* gene.

The present findings towards the end of tumourigenesis complement those from studies on gene expression changes in Eker *versus* wild-type rats during up to two weeks exposure to aristolochic acid or OTA [22]. Cell proliferation was assessed immunohistochemically for proliferating cell nuclear antigen (PCNA) by an anti-PCNA antibody on wax-embedded kidney sections. Differentiated responses suggested that aristolochic acid toxicity was *Tsc2*-independent in both Eker and wild-type rats, whereas that of OTA was more prominently associated with deregulation of mTOR genes in the Eker rats.

For resolving the uncertainty of experimental models for OTA as relevant to human renal tumourigenesis, it is unfortunate that relatively so much scientific effort has focused on only short-term animal experiments and in vitro studies to predict tumour mechanisms, and so little has been devoted to comparing actual tumours in rodents and humans. Several mechanisms that avoid recourse to genotoxicity for OTA have been proposed, e.g., oxidative stress and aberrant mitosis [23]. However, short-term whole animal experiments may not identify influences which fit the requirement for many months of OTA exposure, cultured cells in vitro poorly represent the complexities in kidney, and measurements on kidney tissue are difficult to apply to initiation of a single neoplasm in the outer medulla of only one kidney in a rat. A recent unsatisfactory proposal for aberrant mitosis [24] attempted to apply experimental *in vitro* findings to the *in vivo* situation, but the authors omitted to recognise that most of the OTA in plasma is protein-bound, from which correct extrapolation to their mitotic aberration data from cell-cultures should actually be viewed in the no effect range, negating the perceived relevance to renal tumour formation by OTA. Genotoxicity of OTA is still a matter of debate although the structure of an OTA-DNA adduct has been determined [25]. Finding DNA adducts in tissues is, of course, indicative of OTA exposure, but claims that adducts associated with tumours identify the tumourigen are unsustainable, particularly since adducts have recently been found in rat and human blood [26]. Thus, adducts could reasonably be detectable in all well-vascularised tissues and the extent to which analysis measures adducts actually within the tissue parenchyma is obscure. Notably the concept of OTA-DNA adducts in blood, and disconnection of OTA from genesis of rat testis tumours in the present study, further conflicts with perception of OTA as a cause of human testicular cancer [27,28].

Ultimately, the precise mode of action of OTA as a rat renal carcinogen may remain unresolved, and indeed may not matter if the key genome change (s) can be discovered and compared for relevance with those within the range of human upper urinary tract cancers. Meanwhile, the present findings weaken an assumption that the rat is a valid model for considering OTA as a human carcinogen. It would be interesting to study p-S6 expression in OTA-generated renal tumours in male mice [5] since they are the other possible mammalian model, and also to explore citrinin-induced rat renal tumours (adenomas) [29] since citrinin and OTA have structural and toxicological similarities as nephrotoxins [30].

In the rat, OTA exposure elicits renal accumulation of aneuploid karyomegalic nuclei in the outer medulla where tumours seem to arise. However, any role of these unstable nuclei in carcinogenesis is unclear. It is unfortunate that the few experiments with OTA in primates have not included renal histopathology and so it could be reasonable to assume that the same karyomegaly as is seen in rats might also apply in a primate. However, experimental animals are not always perfect models for humans. Therefore, the contrast between the striking renal histopathological response in the rat to four days of dietary administration of extract of wheat moulded by *Penicillium polonicum*, and the absence of any adverse response in a vervet monkey to 10 days of nasogastric administration of the same extract to a cumulative 5-fold greater dose than in the rat on a body weight basis, [31,32] may be a relevant consideration. 

For human relevance, some regard OTA as a cause of the Balkan endemic nephropathy and its associated tumours of the upper urinary tract, but compelling evidence of exposure and relevant extrapolation from experimental animal toxicology is lacking. Yet, for example, there is precautionary EU legislation concerning tolerable human intake of OTA from food, and necessarily sophisticated analytical monitoring of food components for traces of the toxin. Further immunohistochemical study of the present rat OTA tumours could contribute to debate about relevance of the rat model.

## 4. Experimental Section

### 4.1. Rat and Human Tissues

Rat tissues were in wax blocks archived at Imperial College London mainly during the past decade and mostly from published experimentation [6,8,10,11,12,28,32], with tumour illustration cited in the Supplementary Data Table S1). Similarly archived human tumour tissues were from Denmark (a metastasising renal cell carcinoma [9]) and Romania (County Hospital, Timisoara; four transitional cell carcinomas from cases of Balkan endemic nephropathy [9]); details of their histopathology and DNA ploidy distribution have also been published [9]. A Romanian human angiosarcoma was also used.

### 4.2. Immunohistochemistry

In the Breast Tissue and Data Bank Laboratory, Guy’s Hospital, London, immunostaining was performed on 3 μm thick sections cut from formalin-fixed paraffin- embedded samples. Sections were placed on charged glass slides, dried for 30 min in 42 °C and incubated for no longer than 2 h in an oven at 60 °C. Sections were cut and stained on the same day. Antigen retrieval was performed using citrate buffer (pH 6.0) during 10 min boiling in a microwave oven. Endogenous peroxidase activity was blocked with 3% hydrogen peroxide for 10 min. Immunostaining was performed following the manufacturer’s description using Vectastain Elite ABC kit (Vectastain PK-6101). In addition, avidin/biotin block (Vectastain SP-2001) was applied prior to the primary antibody. Primary polyclonal Phospho-S6 Protein (Ser240/244) (Cell Signaling #2215) antibody in 1:200 dilution was applied to the tissue and incubated overnight at 4 °C. Sections were developed using DAB, counterstained in Gill III haematoxylin, dehydrated and mounted with DPX. Immunostained and H & E stained sections were scanned using the Hamamatsu Nanozoomer and stored on the digital slide server (DSS) in ndpi format. They were reviewed using Digital Images HUB (Slidepath system) for online validation and record. Analyses have essentially been made blinded over several years, with reference only to the study of *Tsc1* expression in mouse [18]. 

In the Pathology Department, County Hospital, Timisoara, formalin-fixed, paraffin-embedded tissue sections of 3–4 μm were mounted on charged slides and dried for 1 h at 56 °C. The sections were deparaffinized in xylene and rehydrated through graded alcohols to water. Endogenous peroxidase activity was quenched with 3% H_2_O_2_, for 5 min, at RT. Heat-induced epitope retrieval method was performed in Tris/EDTA buffer, pH 9, at 97 °C, for 20 min, using a hot water bath. The slides were incubated with the monoclonal mouse antihuman ready-to-use primary antibodies: CD34 class II (DAKO cat. No. IR 632), CD31 Endothelial Cell (DAKO cat. No. IS 610) and D2-40 (DAKO cat. No. IS 072) respectively, for 15 min at RT. For detection the Dako REAL™ EnVision™ Detection System, Peroxidase/DAB+, Rabbit/Mouse (DAKO Code K5007) was used; the visualization was achieved with DAB. Slides were counterstained in haematoxylin, dehydrated, cleared and mounted using permanent mounting medium.

## 5. Conclusions

Phospho-S6 ribosomal protein was consistently expressed in rat renal carcinomas and adenocarcinomas in response to long-term exposure to dietary OTA, in contrast to its absence from adjacent uninvolved kidney and other natural tumours. This correlates with occurrence of this protein in renal neoplasms of the Eker rat strain, and in mice, both with constitutive changes in their tuberous sclerosis complex (TSC) genome which is associated with spontaneous renal tumours. Present findings suggest that OTA carcinogenicity in rats involves changes in the mTOR pathway. However, negative immunohistochemical staining was found for p-S6 in five human renal or upper urinary tract tumours in the present study. Therefore, rat OTA tumourigenesis appears to mimic the contrived genetic change in the Eker rat rather than necessarily to offer a model applicable to predicting human relevance, either to renal cancer in general or the Balkan endemic nephropathy tumours in particular. 

## Supplementary

**Table S1:** Provenance of rat tissue samples, summary of immunohistochemical response, and links to main Toxins manuscript and cited bibliography.

Rat ID	Strain/gender/age at OTA start	OTA dose µg/kg	Weeks On OTA	Latency from OTA to finding tumour	Age (wk) at death	Organ	Tumour type	Histopathology/DNA ploidy distribution	Cited * (Figure in this text)	Immunohistochemistry response
1	DA	250	26	93 w	119	kidney	carcinoma, possibly metastatic from other tumourous kidney	infiltrating (3.2 mm), enveloping glomeruli, not distorting the kidney.	A	Scattered intensely +ve elements
	M								(1A)	
	8w									
						lung	scattered malignant foci	possibly metastatic from contralateral kidney’s carcinoma	A	Scattered intensely +ve elements
									(2D)	
2	DA	250	39	83 w	122 (losing weight)	kidney ‘a’	none recognised initially	-	A	Intense staining of swollen nephron fragment at cortico-medullary junction. Implies early neoplasm.
	M								(1F)	
	8w									
						kidney ‘b’: no tumour apparent	micro-adeno-carcinoma	(2.5 mm), already infiltrating	A	Clear differentiation of tumour from cortex, but not as striking a contrast in staining as in kidney ‘a’
3	SD/F	200	36	61 w	114	kidney, bilateral	adeno-carcinoma (two, small)	erupting tumour, seen in two parts in section. diploid	B, G	Contrasting intense +ve in tumour; some diffuse +ve elements in kidney periphery.
	M								(2C)	
	8w									
						subcutaneous tumour	sarcoma (typical)	diploid	G	-ve, contrasting with kidney and renal tumour of same animal.
									(1H)	
4	SD/F	150	36	61 w	114	mammary	angio-sarcoma in fibro-adenoma	typically sarcomatoid region of tumour.	B	Intense + ve staining in sarcomatoid elements only.
	F								(1E)	
	8w									
						kidneys	none	-	B	Scattered + ve elements in cortex. -ve in medulla and papilla.
5	F344	300	43	46 w	97	kidney	Carcinoma (large)	infiltrating into kidney. aneuploid	G	Contrasting +ve in non-necrotic regions of tumour, -ve in distorted kidney.
	M								(3A)	
	8w									
						testis	typical rat age-related seminomas	diploid	H	-ve in both seminoma and seminiferous tubules
									(1B)	
6	F344	300	91	0	99 (pre-mature; s/c sarcoma)	kidney	carcinoma (large)	infiltrating into kidney		Contrasting +ve of tumour; -ve in all remaining kidney.
	M									
	8w									
7	F344	300	83	0	91	kidney	adeno-carcinoma	compression tumour with some adenoma regions	(1D)	-ve in adenomatous regions of tumour as in renal cortex. Contrasting +ve in carcinoma regions of tumour.
	M									
	8w									
						testis	seminomas	typical of ageing rats	H	-ve
8	F344	300	43	49 w	100 (weight loss, moribund)	kidney (other kidney with major metastasising carcinoma)	carcinoma (tumourous kidney 3g)	tumour infiltrating into and erupting from kidney.		Tumour clearly differentiated from kidney by +ve elements particularly around the tumour periphery, but also scattered throughout and more abundant than in the surrounding kidney.
	M									
	8w									
9	F344	300	93	0	101	kidney	carcinoma	tumourous kidney 8g		Contrasting +ve in tumour.
	M									
	8w									
10	F344	200	102	0	110	kidney	Carcinoma surrounding a cyst	compression tumour distorting kidney near pelvis.	C	Contrasting +ve in tumour; -ve in kidney. Isolated neoplastic focus revealed by intense staining.
	M								(3B)	
	8w									
11	F344	200	105	0	113	kidney	carcinoma (large)	Metastasising. Aneuploid. Infiltrating under capsule.	C,G	Contrasting +ve in non-necrotic regions of tumour; -ve in necrotic tumour and in distorted kidney.
	M								(3D)	
	8w									
12	F344	200	84	0	92	kidney	Adenoma, according to H & E stained histology.	spherical compression tumour (17 mm diameter). Diploid.	C,G	Contrasting +ve in tumour periphery; -ve in stretched kidney cortex. Tumour centre -ve, correlated with necrosis and haematoma.
	M								(4,2B)	
	8w									
13	F344	200	60	0	110	kidney	adenoma	small (6 mm) compression tumour	D	-ve in tumour
	M								(1C)	
	50w									
14	F344	200	35	35 w	120	kidney	micro-adenoma	compression tumour (3 mm)	D	-ve in tumour
	M								(3C)	
	50w									
15	F344	200	35	29 w	114	kidney	micro-adenoma	compression tumour (0.1 mm)	D	-ve in tumour
	M									
	50w									
16	F 344	200	35	38 w	113	kidney	adeno-carcinoma	compression tumour (16 x 12 mm)	D	Diffuse +ve in tumour contrasting with surrounding kidney
	M									
	50w									
17	F344	50	93	0	101	kidney	micro-adeno-carcinoma	(0.5 mm) at cortico-medullary junction; evolving to carcinoma	E	Intense +ve staining of remaining tumour fragment in a nearby section.
	M								(2A)	
	8w									
18	DA	~ 30 thresh-old	30	0	pneu-monia	kidney	-	*-*	A	Faint diffuse differentiation of 3 medullary regions and the cortex
	M								(1G)	
	8w									
19	DA	~ 30 thresh-old	75	0	(weight loss)	kidney	-	*-*	A	Clearer diffuse differentiation of the 4 regions
	M								(1G)	
	8w									
20	SD	-	-	-	10	kidney	none	-	-	-ve
	M									
	-									
21	SD	-	-	-	0 neonatal	kidney (3 animals)	none	-	F	-ve
	-									
	-									

DA, Dark Agouti. SD, Sprague-Dawley. F344, Fischer. SD/F, Hybrid Sprague-Dawley female x Fischer male. * Cited references: A, Mantle 2009 [8]. B, Mantle *et al.* 2009 [10]. C, Mantle *et al.* 2005 [6]. D, Mantle and Nolan 2010 [11]. E, Mantle and Kulinskaya 2010 [12]. F, Mantle 1994 [32]. G, Brown *et al.* 2009 [18]. H, Mantle 2010 [28].

## References

[1] Krogh P., Smith J.E., Henderson R.S. (1991). Porcine Nephropathy Associated with Ochratoxin A. Mycotoxinsand Animal Foods.

[2] The European Food Safety Authority (2006). Opinion of the scientific panel on contaminants in the food chain on a request from the Commission related to ochratoxin A in Food. EFSA J..

[3] Boorman G.A. (1989). Toxicology and Carcinogenesis Studies of Ochratoxin A (CAS No. 303-47-9) in F344/N Rats (Gavage Studies); Technical Report 358.

[4] Castegnaro M., Mohr U., Pfohl-Leszkowicz A., Esteve J., Steinmann J., Tillmann T., Michelon J., Bartsch H. (1998). Sex- and strain-specific induction of renal tumors by ochratoxin A in rats correlates with DNA adduction. Int. J. Cancer.

[5] Bendele A.M., Carlton W.W., Krogh P., Lillehoi E.B. (1985). Ochratoxin A carcinogenesis in the (C57BL/6J x C3H) F1 mouse. J. Natl. Cancer Inst..

[6] Mantle P., Kulinskaya E., Nestler S. (2005). Renal tumourigenesis in male rats in response to chronic dietary ochratoxin A. Food Addit. Contam..

[7] Maher E.R., Aitchison M., Oliver R.T.D., Miles A. (2004). Identification of the Individual at Risk: The Molecular and Clinical Genetics of Renal Cell Carcinoma. The Effective Management of Renal Cell Carcinoma.

[8] Mantle P.G. (2009). Minimum tolerable exposure period and maximum threshold dietary intake of ochratoxin A for causing renal cancer in male Dark Agouti rats. Food Chem. Toxicol..

[9] Mantle P.G., Amerasinghe C., Brown A.L., Herman D., Horn T., Krogh T., Odell E.W., Rosenbaum T., Tatu C.A. (2010). A pilot study of nuclear instability in archived renal and upper urinary tract tumours with putative ochratoxin aetiology. Toxins.

[10] Mantle P.G., Dobrota M., Gillett C.E., Odell E.W., Pinder S.E. (2010). Oncological outcomes in rats given nephrocarcinogenic exposure to dietary ochratoxin A, followed by the tumour promoter sodium barbital for life: a pilot study. Toxins.

[11] Mantle P.G., Nolan C.C. (2010). Pathological outcomes in kidney and brain in male Fischer rats given dietary ochratoxin A, commencing at one year of age. Toxins.

[12] Mantle P., Kulinskaya E. (2010). Lifetime, low-dose ochratoxin A, dietary study on renal carcinogenesis in male Fischer rats. Food Add. Contam..

[13] Maher E.R., Ricketts C.J. (2012). personal communication.

[14] Hasumi Y., Baba M., Ajima R., Hasumi H., Valera V.A., Klein M.E., Haines D.C., Merino M.J., Hong S.-B., Yamaguchi T.P. (2009). Homozygous loss of *BDH* causes early embryonic lethality and kidney tumor development with activation of mTORC1 and mTORC2. Proc. Natl. Acad. Sci. USA.

[15] McDorman K.S., Wolf D.C. (2002). Use of the spontaneous *Tsc2* knockout (Eker) rat model of hereditary renal cell carcinoma for the study of renal carcinogens. Toxicol. Pathol..

[16] Ruggero D., Pandolfi P.P. (2003). Does the ribosome translate cancer?. Nat. Rev. Cancer.

[17] Kasprzak K.S., Diwan B.A., Knonishi N., Misra M., Rice J.M. (1990). Initiation by nickel(11) acetate and promotion by sodium barbital of renal cortical epithelial tumours in male F344 rats. Carcinogenesis.

[18] Brown A.L., Odell E.W., Mantle P.G. (2007). DNA ploidy distribution in renal tumours induced in male rats by dietary ochratoxin A. Exp. Toxicol. Pathol..

[19] Wilson C., Bonnet C., Guy C., Idziaszczyk S., Colley J., Humphreys V., Maynard J., Sampson J.R., Cheadle J.P. (2006). *Tsc1* haploinsufficiency without mammalian target of rapamycin activation is sufficient for renal cyst formation in *Tsc1*^+/−^ mice. Cancer Res..

[20] Kenerson H.L., Aicher L.D., True L.D., Yeung R.S. (2002). Activated mammalian target of rapamycin pathway in the pathogenesis of tuberous sclerosis complex renal tumours. Cancer Res..

[21] Iwenofu O.H., Lackman R.D., Staddon A.P., Goodwin D.G., Haupt H.M., Brooks J.S. (2008). Phospho-S6 ribosomal protein: A potential new predictive sarcoma marker for targeted mTOR therapy. Modern Pathol..

[22] Stemmer K., Ellinger-Ziegelbauer H., Ahr H.J., Dietrich D.R. (2007). Carcinogen-specific gene expression profiles in short-term treated Eker and wild-type rats indicative of pathways involved in renal tumourigenesis. Cancer Res..

[23] Mally A., Dekant W. (2009). Mycotoxins and the kidney: modes of action for renal tumor formation by ochratoxin A in rodents. Mol. Nutr. Food Res..

[24] Czakai K., Muller K., Mosesso P., Pepe G., Schulz M., Gohla A., Patnaik D., Dekant W., Higgins J.M.G., Mally A. (2011). Perturbation of mitosis through inhibition of histone acetyltransferases: The key to ochratoxin toxicity and carcinogenicity?. Toxicol. Sci..

[25] Mantle P.G., Faucet-Marquis V., Manderville R.A., Squillaci B., Pfohl-Leszkowicz A. (2010). Structures of covalent adducts between DNA and ochratoxin A: A new factor in debate about genotoxicity and human risk assessment. Chem. Res. Toxicol..

[26] Pfohl-Leszkiwicz A., Faucet-Marquis V., Tozlovanu M., Peraica M., Stefanovic V., Manderville R. C8-2′-Deoxyguanosine ochratoxin A-adducts and OTA metabolites in biologic fluids as biomarkers of OTA exposure. Proceedings of MycoRed International Conference.

[27] Jennings-Gee J.E., Tozlovanu M., Manderville R., Miller M.S., Pfohl-Leszkowicz A., Schwartz G.G. (2010). Ochratoxin A: *In utero* exposure in mice induces adducts in testicular DNA. Toxins.

[28] Mantle P.G. (2010). Comments on “Ochratoxin A: *In utero* exposure in mice induces adducts in testicular DNA. *Toxins* 2010, *2*, 1428–1424”—Mis-citation of rat literature to justify a hypothetical role for ochratoxin A in testicular cancer. Toxins.

[29] Arai M., Hibino T. (1983). Tumorgenicity of citrinin in male F344 rats. Cancer Lett..

[30] The European Food Safety Authority (2012). Scientific opinion on the risks for public and animal health related to the presence of citrinin in food and feed. EFSA J..

[31] Mantle P.G., McHugh K.M., Fincham J.E. (2010). Contrasting nephropathic responses to oral administration of extract of cultured *Penicillium polonicum* in rat and primate. Toxins.

[32] Mantle P.G. (1994). Renal histopathological responses to nephrotoxic *Penicillium aurantiogriseum* in the rat during pregnancy, lactation and after weaning. Nephron.

